# A streamlined search technology for identification of synergistic drug combinations

**DOI:** 10.1038/srep14508

**Published:** 2015-09-29

**Authors:** Andrea Weiss, Robert H. Berndsen, Xianting Ding, Chih-Ming Ho, Paul J. Dyson, Hubert van den Bergh, Arjan W. Griffioen, Patrycja Nowak-Sliwinska

**Affiliations:** 1 Institute of Chemical Sciences and Engineering, Swiss Federal Institute of Technology (EPFL), Lausanne, Switzerland; 2 Angiogenesis Laboratory, Department of Medical Oncology, VU University Medical Center, Amsterdam, The Netherlands; 3 Med-X Research Institute, School of Biomedical Engineering, Shanghai Jiao Tong University, Shanghai, China; 4 Department of Mechanical and Aerospace Engineering, University of California, Los Angeles, USA

## Abstract

A major key to improvement of cancer therapy is the combination of drugs. Mixing drugs that already exist on the market may offer an attractive alternative. Here we report on a new model-based streamlined feedback system control (s-FSC) method, based on a design of experiment approach, for rapidly finding optimal drug mixtures with minimal experimental effort. We tested combinations in an *in vitro* assay for the viability of a renal cell adenocarcinoma (RCC) cell line, 786-O. An iterative cycle of *in vitro* testing and s-FSC analysis was repeated a few times until an optimal low dose combination was reached. Starting with ten drugs that target parallel pathways known to play a role in the development and progression of RCC, we identified the best overall drug combination, being a mixture of four drugs (axitinib, erlotinib, dasatinib and AZD4547) at low doses, inhibiting 90% of cell viability. The removal of AZD4547 from the optimized drug combination resulted in 80% of cell viability inhibition, while still maintaining the synergistic interaction. These optimized drug combinations were significantly more potent than monotherapies of all individual drugs (p < 0.001, CI < 0.3).

Clinical experience has shown that, in many cases, the use of a single targeted agent for the treatment of cancer does not provide long-term responses^1^. This limitation is largely due to (i) the complexity of cancer cell signaling pathways, which are redundant and adaptive^2^, (ii) tumor heterogeneity^3^, and (iii) the development of drug resistance^4^,^5^. Indeed, many improvements in cancer therapy have emanated from the use of drug mixtures, where the inhibition of parallel pathways with non-cross-resistant drugs improves treatment efficacy and reduces the occurrence of drug resistance^6^. This approach has already been strongly investigated in the use of combination chemotherapy for cancer^7^,^8^. Yet, there are many challenges to optimizing drug combinations. These include the immense search space represented by the amount of available drugs used at multiple concentrations and the limited number of combinations that can be tested due to limitations of time and resources, in particular in a clinical setting. Thus, new and fast methods are required to identify optimal drug combinations. A sufficiently rapid screening method could be used to develop personalized medicine, where optimal combinations of targeted drugs are screened for a particular patient following molecular profiling of a tumor biopsy.

Several methods to optimize drug combinations have already been investigated, including cell modeling techniques^9^,^10^, systematic based searches^11^ and optimizations based on deterministic^12^ and stochastic search algorithms^13^,^14^. Among these methods, the feedback system control (FSC) technique is a recently developed platform, which has been successfully applied to optimize drug combinations for various complex biological systems, including the inhibition of viral infection^15^, the maintenance of human embryonic stem cells^16^ the differentiation of mesenchymal stem cells^17^, and the inhibition of tumor angiogenesis^18^. The latter study describes the use of a stochastic algorithm that searches for drug combinations that optimize a desired cellular output. Thus, the drug combination optimization procedure is phenotypically driven and the actual optimization process does not necessarily require mechanistic information to identify the optimal drug combinations^19^.

In the present study, we introduce a streamlined route to drug combination optimization and implement this novel approach in the optimization of a multi-drug combination inhibiting the viability of the human renal adenocarcinoma cell line 786-O. The selection of this cell line was motivated by the fact that renal cell carcinoma (RCC) is generally chemo- and radio-therapy resistant, and its clinical treatment is therefore largely dependent on the use of targeted agents^20^. Ten drugs targeting a broad spectrum of pathways reported to play a role in RCC progression were selected. After only three rounds of experimental search efforts, we identified an optimal drug combination containing axitinib^21^; erlotinib^22^,^23^, dasatinib and AZD4547. This drug combination induced the specific and synergistic^24^ inhibition of 786-O cell viability, while having a reduced effect on non-malignant cell types.

## Results

### Drug optimization by the model-based streamlined feedback system control system (s-FSC)

**Figure 1:**
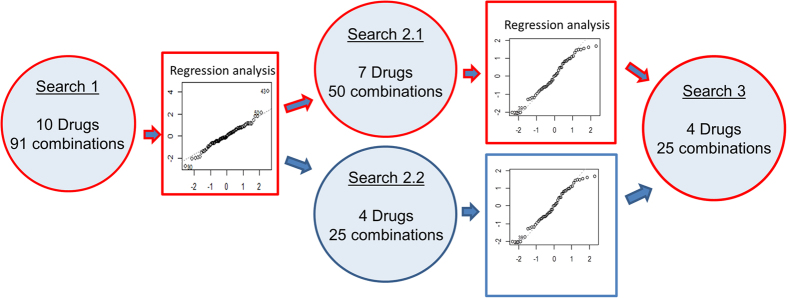
Overview of the streamlined s-FSC technique used for *in vitro* drug combination optimization showing the three search steps used. The procedure starts with the first search where the interactions of the initial set of drugs were investigated by testing 91 drug combinations for cell viability inhibition. The results are analysed using a second-order linear regression analysis that enables the elimination of the least significant compounds. In the next step (Search 2.1) the combinations of all remaining drugs or, in parallel, of the four most effective compounds (Search 2.2) identified in Search 1, were analysed. Searches 2.1 and 2.2 identified a group of four compounds, which appeared to show the strongest single drug actions, as well as synergistic interactions. A third search was therefore performed to test the interaction of these four compounds in combination. The highlighted pathway corresponds to the final s-FSC technique scheme.

The s-FSC technique is based on an iterative cycle, where drug combinations are tested *in vitro* and analyzed in a series of ‘search rounds’, each resulting in the development of a second-order linear regression model (Fig. 1). The analysis of the regression coefficients associated with these models allows for the progressive elimination of ineffective or antagonistic drugs from the selection and for the optimization of the dose ratio of the final drug combination.

The optimization process was initiated with ten targeted agents, each affecting major signalling pathways known to be involved in the growth and progression of renal cell adenocarcinoma (Supplementary Figure 1). This drug set included: 1. axitinib, (VEGFRs, PDGFR and c-kit inhibitor^21^); 2. erlotinib (EGFR inhibitor^22^,^23^); 3. RAPTA-C (a histone inactivator^25^,^26^,^27^), 4. BEZ-235 (mTOR/PI3K/AKT^28^ inhibitor); 5. Volasertib (PLK- polo-like kinase^29^ inhibitor); 6. dasatinib (BCR/Abl, Src and c-kit inhibitor, not affecting EGFR or Her2^30^); 7. VX-680 (Aurora A, B, C^31^ inhibitor); 8. U-104 (an inhibitor of carbonic anhydrase IX/X^32^,^33^); 9. AZD4547 (FGFR1-3 inhibitor^34^) and 10. crenolanib (specific PDGFR inhibitor^35^).

For each of these compounds, broad dose-response curves were built measuring their efficacy in the inhibition of 786-O cell viability (Supplementary Figure 2). These curves were used to identify doses of each compound to be used in the optimization. An EC_50_ value of each drug was estimated based on fitting the *in vitro* data with a linear fit (Table 1). The highest dose, referred to as dose 2, corresponds to effective dose (ED) of approximately 25% inhibition versus control (ED_25_). Dose 1 was selected to have an efficacy between EC_5_ and EC_10_ and dose 0 signified that no drug was present (refer to Supplementary Information for more details).

**Figure 2:**
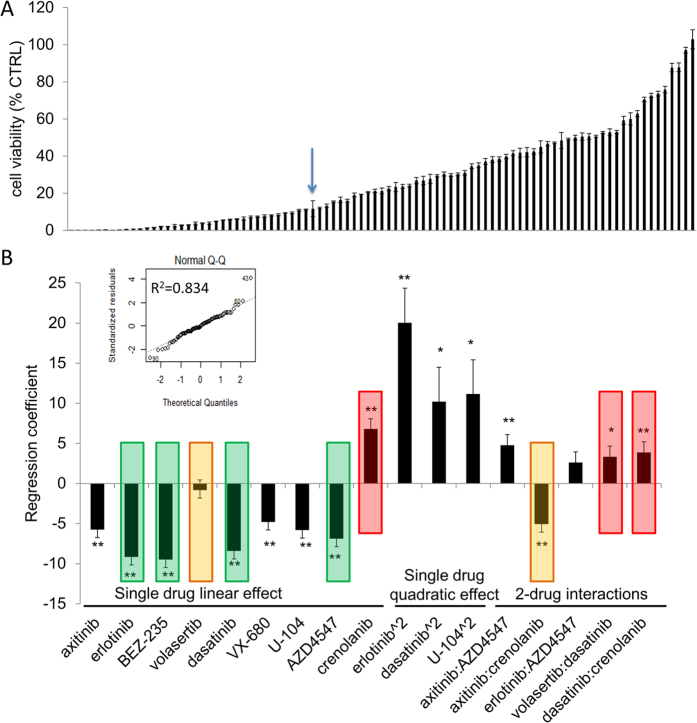
Optimization of the inhibition of cell viability in Search 1 and data modeling. (**A**) Efficacy of the 91 drug combinations on cell viability inhibition. The arrow indicates the drug combination, which contains all ten drugs at dose 2. (**B**) The coefficients from the second order linear regression analysis applied to data obtained in Search 1. The coefficients of the second-order linear regression fitted to the experimental data from Search 1. Single drug second-order terms are denoted as being raised to the power of two (‘drugname^2′). The results from this second-order stepwise linear regression model allow the least effective compounds to be eliminated (red highlighted bars indicate contributions of volasertib and crenolanib which are not inhibitory to cell viability, bars highlighted in orange indicate minimal contributions or cell inhibitory contribution that are overpowered by other antagonistic interactions). RAPTA-C showed no significant interactive effects with any of the compounds and does not appear in the step-wise model. The four most promising compounds (erlotinib, BEZ-235, dasatinib and AZD4547) are highlighted in green. The most significant effects are seen in the first order terms. Inset: linear fit of modelled data showing theoretical quartiles versus standardized residuals (R^2^ = 0.834). The significance is indicated by asterisks with **p < 0.01 and *p < 0.05.

The drug combinations to be tested *in vitro* were defined based on a ‘design of experiment approach’ (DOA). Designed matrices referred to as ‘orthogonal array composite designs’ (OACD) (see Supplementary Information) were used. The data obtained using these matrices allowed for the generation of regression models which provide non-aliased estimates of all first order terms, as well as estimates of the two-drug interaction effects and second order single drug effects, after testing only a minimal number of data points (Fig. 1). Search 1 was performed using the ten compounds at the three doses defined above by testing ninety-one drug combinations based on an OACD matrix^36^ provided in Supplementary Table 1. The efficacy of cell viability inhibition for all combinations is provided in Fig. 2A. The combination containing all ten compounds at dose 2 resulted in 89% inhibition of cell viability (blue arrow in Fig. 2A). Of note, this combination was outperformed by various combinations containing fewer drugs.

The data points obtained from Search 1 were used to generate a second-order stepwise linear regression model (Fig. 2B). The regression coefficients for each specific compound correspond to its impact on overall drug mixture efficacy, i.e. a negative regression value indicates that increasing the dose of that drug increases the inhibition of cell viability. A second-order regression model was applied, as it has previously been shown that higher terms are generally much smaller than first- and second- order terms^37^,^38^. Therefore, regression coefficients describing single linear drug effects, two-drug interaction terms and single drug quadratic effects are provided in this model (Fig. 2B).

Compounds with the lowest negative regression coefficients were the least active in combination and were therefore eliminated, in this case volasertib, crenolanib and RAPTA-C (Fig. 2B). The modeling results were verified by analyzing the composition of the most effective combinations identified, and revealed erlotinib, BEZ-235, dasatinib and AZD4547 as the most frequently present compounds in the ten most effective combinations (Supplementary Table 1). In order to investigate the possibility of directly identifying the main control parameters (or drugs) from the initial ten drug interaction model, Search 2 was comprised of two parallel steps: Search 2.1 with all seven remaining compounds as compared to Search 2.2 containing only the four most dominant compounds, as shown in Supplementary Tables 2 and 3.

**Figure 3:**
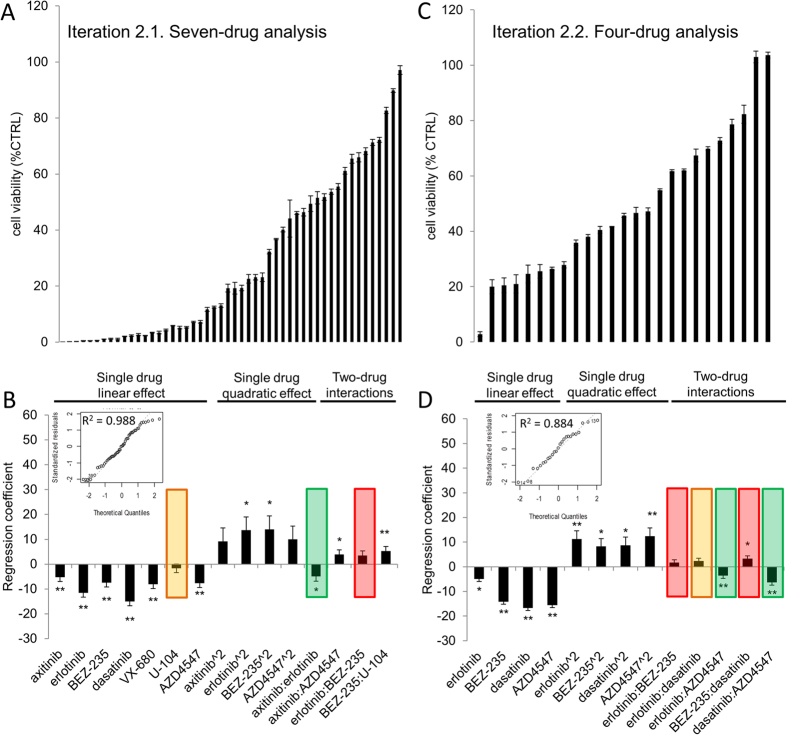
Effect of the drug combinations for the inhibition of cell viability and data analysis of Search 2.1 (**A, B**) and Search 2.2 (**C, D**). The performance of the 50 drug combinations tested for inhibition of 786-O cell viability for the set of 7-drug in Search 2.1 (**A**) and the set of 25 drug combinations of 4-drugs investigated in Search 2.2 (**C**). Regression coefficients from the second-order stepwise linear regression model are provided based on the data obtained from Search 2.1 (**B**) and Search 2.2 (**D**). The results allow the least effective compounds to be eliminated. Red is used to highlighted drug contributions that do not help to inhibit cell viability, orange bars are used to indicate minimal contributions and green highlights synergistic drug interactions. The insets in B and D show the linear fit of modelled data with theoretical quartiles versus standardized residuals and the corresponding R^2^-values for each model. Significance is indicated by asterisks, **p < 0.01 and *p < 0.05.

The efficacy of the fifty drug combinations tested in Search 2.1 are provided in Fig. 3A. Regression coefficient analysis presented in Fig. 3B shows only minimal single drug contributions from U-104 (not significant, indicated in orange), as well as antagonistic interactions with BEZ-235 (**p < 0.01, indicated in red, Fig. 3B). VX-680 showed a relatively strong single drug effect (**p < 0.01); however, this drug was less potent as a single drug than erlotinib and dasatinib and showed no significant interactions with the other compounds.

In Search 2.2, only the most effective 4 drugs, identified by modeling of the data in Search 1, were examined (Fig. 3C). Regression analysis identified synergies between axitinib and erlotinib (*p < 0.05), erlotinib and AZD4547 (**p < 0.01), as well as dasatinib and AZD4547 (**p < 0.01). Antagonistic interactions were identified between BEZ-235 and erlotinib (non-significant in Searches 2.1 and 2.2, Fig. 3B,D in red), as well as between BEZ-235 and dasatinib (*p < 0.05 Search 2.2, Fig. 3C in red).

Taken together, these results suggest that the four most relevant compounds were axitinib, erlotinib, dasatinib and AZD4547. Moreover, this proves that Search 2.1, but not 2.2, was crucial to correctly identify these compounds.

**Figure 4:**
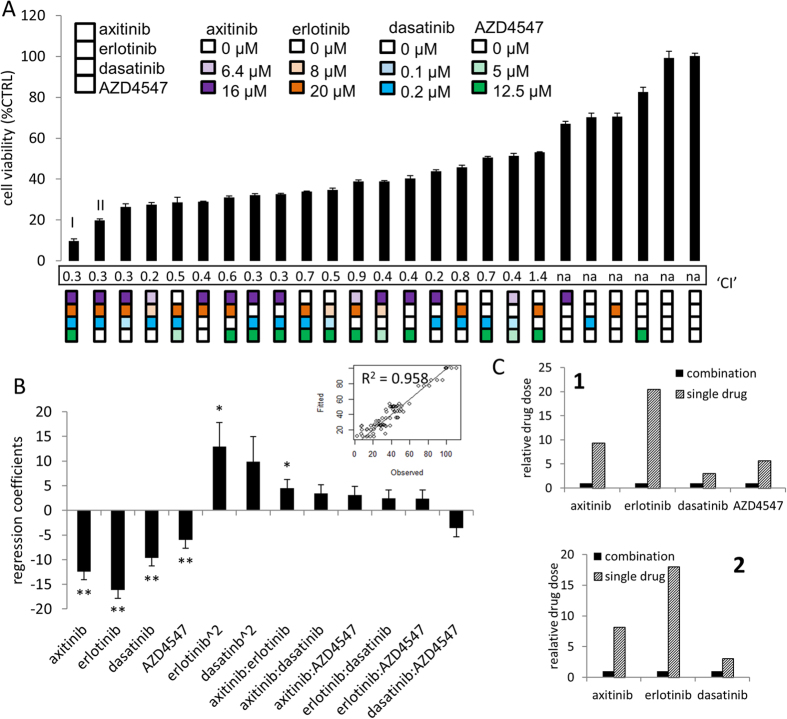
Identification of the optimal drug combination for the inhibition of 786-O cell viability based on results from Searches 1 and 2.2. (**A**) Cell viability inhibition efficacy of each drug combination tested in Search 3. The square icons present the specific combinations, where each position in the square and color corresponds to a specific drug (i.e. the top square and color purple indicate axitinib and the concentrations (μM) of each compound are represented by the different patterns). The most promising combinations, labeled **I** and **II**, represent a mean of at least three independent experiments, with three replications each, and error bars represent the SEM. (**B**) Regression coefficients from the fit with the second-order stepwise linear regression model based on the data of Search 3. Insets in B show the linear fit of modelled data with theoretical quartiles versus standardized residuals and the corresponding R^2^-values for each model. (**C**) Relative drug dose required to obtain an equivalent level of efficacies as seen for the combinations **I** and **II** when drugs are applied in combination or individually.

In order to confirm this selection, we performed the final experimental set, Search 3 (Supplementary Table 3). The efficacy on cell viability inhibition of all 25 drug combinations tested in the third search is summarized in Fig. 4A with the most effective drug combination indicated as (**I** and **II**). In addition, the combination index (‘CI’) for each drug combination is provided indicating synergistic interactions when CI < 1. The data obtained in this search was used to generate a linear regression model and the corresponding regression coefficients are presented in Fig. 4B. These regression coefficients showed significant single drug linear effects for all compounds. This is in agreement with the experimental results showing the most effective combination containing all four compounds. Optimized drug combinations allowed for a significant reduction in individual drugs doses required as compared to the individual drug doses that would be needed to obtain the same overall efficacy (Fig. 4C). Drug combination **I** allowed for reduction of 9-, 20-, 3- and 6-fold compared to theoretical drug doses required for single drugs to achieve 90% cell viability inhibition.

### Activity of optimal combinations in other cell types and in cell migration- and apoptosis assays

**Figure 5:**
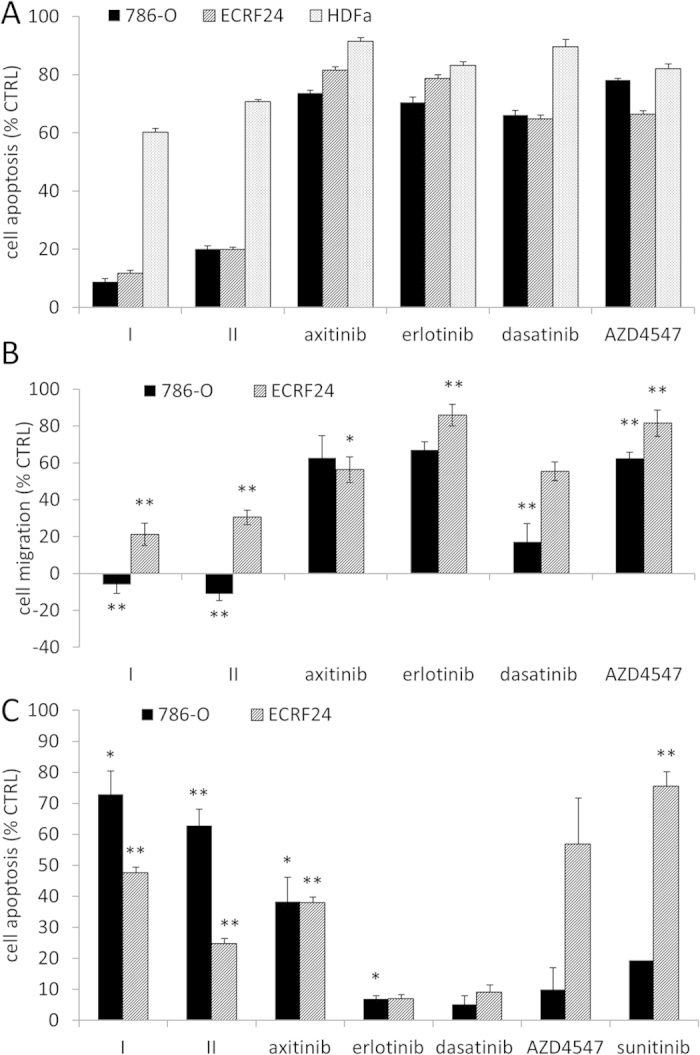
Summary of the data used to validate the optimized drug combinations I and II. (**A**) The effects of the most promising drug combinations **I** and **II**, refer to Fig. 4A, and their corresponding monotherapies were tested on the viability of the following non-malignant cell lines: immortalized human endothelial cells (ECRF24), and adult human dermal fibroblasts (HDFa). (**B**) Migration inhibition of ECRF24 and 786-O cells with corresponding monotherapies. (**A,B**) Values represent the mean of at least 2 independent experiments, with 3 replications each. Error bars represent the SEM. (**C**) The effects of individual compounds and combinations on ECRF24 and 786-O cell apoptosis induction. Values represent the mean of at least 2 independent experiments and error bars represent the SEM. Asterisks represents significance tested by the student’s t-test, *p < 0.05 and **p < 0.01.

The best-optimized drug combinations, **I** and **II**, were further tested for cell viability inhibition in ECRF24 human endothelial cells and in adult human dermal fibroblasts (HDFa, Fig. 5). The optimized combinations are significantly more potent at inhibiting cell viability than all of their corresponding monotherapies in 786-O cells (p < 0.001), showing strong synergistic activity (CI < 0.2), as well as in ECRF24 cells (Fig. 5A). The sensitivity of adult human dermal fibroblasts (HDFa), however, was reduced as compared to these cell lines.

The mobility of 786-O cells was significantly inhibited by combinations **I** and **II**, as compared to the control (and as compared to all monotherapies for combination **II**). A similar but less pronounced response was observed for ECRF24 cells (Fig. 5B). Cell death was demonstrated to occur through apoptosis. An enhanced number of 786-O cells exposed to combinations **I** and **II** underwent apoptosis. Similarly, apoptosis induction was significantly stimulated by combinations **I** and **II** in ECRF24 cells. However, these cells were sensitive to induction of apoptosis by exposure to axitinib and AZD4547 as well. Sunitinib (20 μM) was used as a positive control in this assay^39^, known to efficiently induce endothelial cell apoptosis, Fig. 5C.

## Discussion

In the study presented here, a feedback system control methodology, termed the s-FSC technique, is presented. Using this technique, it was possible to identify optimized drug combinations after testing only 191 drug combinations, representing significantly less experimental effort than the total 60,000 possible drug combinations (represented by testing all combinations of ten drugs at three doses). In the different approaches used to determine optimal drug combination there is a trade-off between estimation efficiency and test size. Geva-Zatorsky *et al.*^40^ reported a method that required testing 3000 drug combinations to optimize 10 drugs at 8 doses. Using our protocol 435 drug combinations would need to be tested to identify the optimal drug combination. This difference is because only three doses are needed for the s-FSC approach as the number of tests needed is essentially related to the number of drugs and is linearly proportional to the dose levels. Moreover, number of required test points does not proportionally increase with the number of drugs involved in a study. Therefore, even with a relatively larger search space, the s-FSC method is still practical and cost effective.

The s-FSC technique tests statistically designed drug combinations in cell bioassays and paints the bio-complex system with stepwise linear regression models in order to rapidly locate a sub-group of the candidate drugs that provide the most synergistic outcome with minimal experimental data. This systematic search for the best drug combination from a broad array of anti-cancer compounds allowed, for the first time, the identification of optimal drug combinations in only three search steps. The optimization was performed starting with ten targeted compounds acting on distinct signaling pathways of malignant cells. The anti-cancer potential of drug combinations was assessed based on the inhibition of cell viability in cultured human renal cell adenocarcinoma (RCC) cell line, 786-O. This approach provides a practical, non-high-throughput method for large-scale drug combination optimization.

A major advantage of s-FSC is that it allows for the rapid analysis of many different multi-drug combinations. This technique thus allows for a vast increase in the speed and reduction in the complexity of drug mixture optimization compared to the first generation of FSC technique (FSC.I)^18^. The FSC.I implements a stochastic search algorithm that, via an iterative approach of testing a small number of drug combinations in each search, allows for progressive optimization of the drug combination in 10–15 iterations. Since FSC.I optimizes drug combinations iteratively, the accumulative amount of time to apply FSC.I can still be relatively long. s-FSC aims to identify the optimal drug combinations in only 3 rounds of experiments. Although FSC.I has been successfully applied in various biological systems and reduces experimental effort typically by >90%, the total number of experiments required is still quite high^18^.

Mathematical and statistical tools, i.e. a design of experimental approach, were implemented in order to develop accurate descriptive models of cell viability in response to the applied drug combinations. These models included individual drug (first and second order) and drug-drug interaction terms. Drug-drug interaction terms may significantly influence the overall cellular response to a drug combination and help guide the search to identify an optimal three-drug combination. Subsequent testing of these optimal combinations in *in vitro* assays for other cell processes, i.e. cell mobility and induction of apoptosis, also revealed synergistic activities.

At the drug discovery and drug design stage, the drug-drug interactions are minimized for the purpose of reducing the antagonistic effect and hence giving more flexibility during clinical applications. However, non-linear drug-drug interactions may take place through inter-connected biological pathways, even if chemical drug-drug interactions are rather weak. Therefore, a linearly additive approach may be good for certain drugs but not for others. For example Geva-Zatorsky *et al.*^40^ examined the effects of two-drug combinations of 13 drugs on the levels of 15 proteins in human cells and showed that the protein dynamics in combinations can be predicted based on the weighted sum (linear superposition) of individual drug responses. When studying the two-drug interactions, one out of the 13 compounds (a PI3K inhibitor) was shown to not follow the linear superposition rule, a behavior for which the authors could find no explanation. Additionally, Pritchard *et al.*^41^ investigated the mechanism of drug combinations using ‘RNAi-signatures’ in lymphoma cells infected with one of 29 distinct retrovirally expressed shRNAs targeting different checkpoint kinases or cell death regulators. They showed that while the synergistic drug combinations take on the mechanisms that resemble the actions of one of the single drugs (indicating that one agent is enhancing the effect of the other agent), the profile of additive drug combinations act as an average of the single drug components. Weighted average models are used to predict the response of these additive drug combinations and are compared to the measured values, showing relatively good correlations between predicted and measured values of two-drug or four-drug combinations. The authors note a “statistically significant difference in model fit between combinations that acted synergistically and those that acted additively”, and indicate that there is a relatively poor correlation between the predicted and measured values in the synergistic drug combinations. These results support the idea that the shRNA profile of additive drug combinations can be sufficiently predicted without the including non-linear effects, however does not explain the lack-of-fit seen in the synergistic drug combinations, which should also theoretically be explainable by a weighted average model even if the profile resembles one drug’s activity more strongly than the other’s. It is important to note, that neither of these studies examined the input-output response of drug combinations on overall cell fate, which may include non-linearity not present when directly measuring protein levels or shRNA signatures.

In previous studies^15^ we examined the drug interactions between several antiviral drugs on a Herpes Simplex Virus type 1 (HSV-1) model. While some of the individual drugs are effective in viral inhibition, none of them alone could eradicate the viral infection. When combined, at much lower doses, optimal drugs combinations would eliminate the viral infection. This fact indicates that drug interactions, although not existing in all cases, do contribute significantly to better efficacy and lower dose related adverse effects.

Various other groups have also described the response surface of cells to drug combinations as “highly nonlinear”^42^,^43^ where “sometimes nonlinear curve fitting is desirable or actually required”^44^, for example the use of a polynomial fit for the response of a combination of anesthetic drug interactions^45^.

RCC was selected as the targeted indication in this study, as the clinical treatment of this cancer type largely relies on the use of targeted agents^20^. Side-effects and acquired drug resistance^46^ tend to limit the use of targeted agents for cancer therapy. Due to the frequent use of targeted agents for this indication and their limited long-term efficacy, it is of considerable clinical value to develop optimized combination therapy strategies. It should be emphasized that although the current study focuses on new treatment approaches for RCC, it is expected that the s-FSC approach can be applied to other cancer types.

As presented in Fig. 1, our technique was comprised of three search steps starting from the initial multi-drug set and leading to the selection of the final synergistic subset of drugs. Search 2, performed in two parallel steps, revealed that the direct selection of drugs based on Search 1, as tested in Search 2.2, did not lead to the optimal solution. This may be due to the fact that the presence of other drugs in the mixture may screen important interactions. Therefore, Search 1, 2.1 and 3 are the only required steps for an effective s-FSC design (Fig. 1, highlighted path). These results indicate that it may generally be safer to only eliminate a few drugs per round, while performing more cycles of testing.

s-FSC enabled the identification of the optimal drug combinations, in which the dose of each compound was significantly reduced as compared to the theoretical individual drug doses require to obtain the same efficacy. In the optimal combination **II**, individual drug doses were reduced by 8-, 18- and 2.8-fold, for axitinib, erlotinib and dasatinib, respectively. Moreover, many of the compounds tested were unable to attain an equivalent level of cell viability inhibition when applied as single agents even at very high doses.

We also show that other cellular processes are significantly influenced by the optimal drug combinations. Both 786-O cell mobility inhibition and apoptosis induction were more pronounced, as compared to individual drugs. Since RCC is a highly vascularized disease, we also confirm that the best-optimized mixtures are highly effective in ECRF24 proliferation and mobility inhibition, as well as apoptosis induction. This implies that in a clinical environment this treatment would lead to dual-targeting of both the cancer cell- and endothelial cell compartments.

s-FSC technique could be used to develop prognostic markers in cancer patients both in tumor and adjacent non-tumor tissues. This “co-optimization” may potentially lead to decreased side-effects. Li *et al.*^47^ recently reported on a bottom-up approach used to develop prognostic markers in hepatocellular carcinoma (HCC) patients both in tumor and non-tumor tissues. This led to successful development of prognostic gene signatures with genes that associated with overall survival of HCC patients. Moreover, many prognostic gene signatures identified in this study turned out to be druggable targets that have been indicated by approved drugs but not linked to HCC, leading therefore to drug-repurposing.

Drug combinations will become increasingly important to improve the therapeutic effects of drugs and minimize drug toxicity and drug-induced resistance. s-FSC overcomes the limitations of FSC.I and could be used to personalize combination therapy, performing a screen on freshly isolated tumor cells. It may even be feasible to optimize the combination therapy during the course of treatment, thus offering a unique approach to personalized medicine. The study presented here provides a proof-of-concept that improved therapeutic outcomes may be possible through the identification of drug combinations with selective activity towards a selected cancer type.

## Methods

### Drug acquisition

Axitinib and erlotinib were purchased from LC laboratories (Woburn, MA, USA), Sutent^®^ (sunitinib) from Pfizer Inc. (New York, NY, USA) and BEZ-235 from Chemdea LLC (Ridgewood, USA). RAPTA-C was synthesized and purified as described previously^48^. Volasertib, VX-680, U-104, AZD4547 and crenolanib were purchased from Selleck Chemicals (Houston Texas, USA). Dasatinib was purchased from Fluorochem Ltd (Derbyshire, UK). Compounds were dissolved in DMSO and stored at −20 °C for short term use or at −80 °C for storage up to six months with the exception of BEZ-235 which was dissolved in DMSO and stored at + 4 °C and RAPTA-C which was freshly dissolved in DMSO directly before use. The maximum DMSO concentration for any combination was controlled in each experiment to verify its lack of activity in cell assays.

### Cell culture and maintenance

786-O (human renal cell adenocarcinoma) cells were maintained in DMEM cell culture medium supplemented with 1% of antibiotics (penicillin/streptomycin, Life Technologies, Carlsbad, California, USA) and 10% heat-inactivated bovine calf serum (Sigma-Aldrich, St. Louis, USA). Immortalized human vascular endothelial cells (ECRF24) were maintained in medium containing 50% DMEM and 50% RPMI-1640 cell culture medium supplemented with GlutaMAX™ (Gibco, Carlsbad, USA) supplemented with antibiotics and bovine calf serum as above. ECRF24 cells were cultured on 0.2% gelatin coated surfaces. Adult human dermal fibroblast (HDFa) cells were maintained in DMEM, supplemented as mentioned above. Human peripheral blood mononuclear cells (PBMC) were freshly isolated as previously described^49^.

### Cell viability, migration, and apoptosis assay

Cell viability and migration assays were performed as previously described^25^. Cells were seeded in a 96-well culture plate at a density of 1 × 10^4^ cells/well and allowed to attach overnight. Subsequently medium was removed and single drugs or premixed drug combinations were administered. Cells were incubated with drugs for 72 h. Drug dilutions were prepared by serial dilutions and mixtures were prepared by adding the corresponding drugs to medium at the doses specified in Table 1. The cell viability assay was quantified based on luminescence using the CellTiter-Glo luminescent cell viability assay (Promega, Madison, WI, USA).

For the 786-O and ECRF24 migration assays, cells were seeded in 96-well cell culture plates at a density of 30 × 10^3^ cells/well (0.2% gelatin coated for ECRF24 cells). Cells were left overnight to form a confluent monolayer, and, at 24 h after seeding, a scratch wound was made in the cell monolayer using a sterile scratch tool (Peira Scientific Instruments, Beerse, Belgium). Drugs were premixed and administered as done for the proliferation assays. Images were automatically captured on a Leica DMI3000 microscope (Leica, Rijswijk, Netherlands) at 5× magnification with Universal Grab 6.3 software (DCILabs, Keerbergen, Belgium). Scratch sizes were assessed at t = 0 h and t = 7 h using Scratch Assay 6.2 (DCILabs), and analyzed as a percent closure of the initial scratch wound and presented as a percentage of the control well closure.

Apoptosis assays were performed as previously described^50^. 786-O and ECRF24 cells were seeded in a 24-well plate (40 × 10^3^ cells/well) and left overnight to attach. At 24 h after seeding, medium was removed and new medium or drugs were added. Cells were allowed to grow in the presence of drugs for an additional 72 h. After 72 h cells were harvested by trypsinization and incubated with propidium iodide (PI) (20 μg/ml) in buffer containing 2.5 μM citric acid, 45 μM Na_2_HPO_4_ and 0.1% Triton-X100, (pH 7.4, 20 min, 37 °C). Analysis was performed with a FACSCalibur (BD Biosciences) in the FL2 channel and apoptotic cells were defined as having subG1 DNA staining. See Supplementary Dataset 2 for raw data.

### Data analysis and modeling

Drug combinations are tested based on a statistical design of experiment approach called orthogonal array composite design (OACD). The design contained both a two-level factorial or fractional factorial design and a three-level orthogonal array^36^ (Supplementary Table 1–3). Regression analysis was performed in R statistical software (R developmental core team) based on a stepwise linear regression model with the following form:
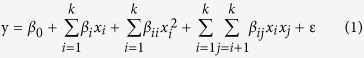
where β_0_, β_i,_ β_ii_ and β_ij_ are the intercept, linear, quadratic and bilinear (or interaction) terms, respectively; γ is the response variable (i.e. cell viability as percent of control); x_i_ and x_j_ are independent variables (i.e. drug combinations at designated doses); ε is an error term with a mean equal to zero^51^. Second-order linear regression models were generated using the data obtained from each search round. Data was modeled using coded concentration values and data was not transformed. See Supplementary Dataset 1 and 3 for raw data.

### Statistical analysis

Unless otherwise stated, values are given as mean values ± standard deviation. Data are represented as averages of independent experiments. Statistical analysis was performed using a two-sided student’s t-test *p < 0.05, and **p < 0.01 were considered statistically significant. Drug combination synergy was quantitatively analyzed using the CompuSYN software^24^. The ‘combination index’ (CI) value calculated for each drug combinations indicated synergistic interactions (CI < 0.8), additive effects (CI = 0.8–1) or antagonism (CI > 1). Additionally, synergy was assessed based on an efficacy gain calculated for each drug combination. This was determined by calculating the difference between the actual efficacy of each combination achieved *in vitro* and the theoretical efficacy of each combinations calculated as the additive effect of each of the corresponding single drug efficacies depending on fractional effect analysis^52^.

## Additional Information

**How to cite this article**: Weiss, A. *et al.* A streamlined search technology for identification of synergistic drug combinations. *Sci. Rep.* **5**, 14508; doi: 10.1038/srep14508 (2015).

## Supplementary Material

Supplementary Information

Supplementary Dataset 1

Supplementary Dataset 2

Supplementary Dataset 3

## Figures and Tables

**Table 1:** Concentrations (μM) of the coded doses (0, 1 and 2) used in drug combination optimization for each compound, as well as the EC_50_ value of each compound on the cell viability inhibition assay of 786-O cells.

Dose (μM)Compound	0	1	2	EC_50_*
axitinib	0.0	6.4	16.0	76
erlotinib	0.0	8.0	20.0	>100
RAPTA-C	0.0	300.0	750.0	>750
BEZ-235	0.0	0.4	1.0	2.4
volasertib	0.0	0.2	0.5	1.1
dasatinib	0.0	0.1	0.2	0.3
VX-680	0.0	6.0	15.0	31
U-104	0.0	25.0	62.5	200
AZD4547	0.0	5.0	12.5	38
crenolanib	0.0	2.0	5.0	12

^*^Values estimated based on linear regression fit.
