# Supplementary material for: A streamlined search technology for identification of synergistic drug combinations
Source: Sci Rep. 2015 Sep 29;5:14508. doi: 10.1038/srep14508 (PMC4586442; doi:10.1038/srep14508)
Supplement: Supplementary Information [file srep14508-s1.doc]

**Supplementary Information**

**A streamlined search technology for identification of synergistic drug combinations**

Andrea Weiss^1,2^, Robert H. Berndsen^1^, Xianting Ding^3^*, Chih-Ming Ho^4^, Paul J. Dyson^1^, Hubert van den Bergh^1^, Arjan W. Griffioen^2^, Patrycja Nowak-Sliwinska^1,2^*

**Contents**

**Supplementary Information**

**Supplementary Figures**

**Supplementary Tables**

**Supplementary References**

**Supplementary Information**

*Design of experiment technique and orthogonal array composite designs*

The design of experiment approach (DOA) technique is frequently used in many fields of research in order to optimize processes, such as chemical reactions. In DOA, the process being optimized is treated as a ‘black box’ with various input and output variables. By performing multiple experiments with different input parameters, one can compare different processes, define the most influential input parameters to the system, and even optimize the system response by more in depth analysis using response surface modeling. DOA can facilitate this by maximizing the information you can obtain from a given number of experiments based on the desired goal. This is achieved by testing combinations of input parameters based on design matrices. These matrices define the data points that need to be tested in order to generate regression models that can accurately describe the terms and interactions of interest.

In the study presented here, we use a special type of design matrix referred to as orthogonal array composite designs (OACD)[^1^](#_ENREF_1). These matrices are similar to central composite designs (CCD) or small composite designs (SCD) as they all use two-level factorial or fractional factorial designs as the cube points of the design, however they differ based on the additional points added to the design. OACDs use runs from a three-level orthogonal array as the additional data points. This unique combination of a two-level fraction factorial design with a three-level orthogonal array generates a resolution IV design matrix that can be used to screen for the most influential factors in the system based on accurate estimations of each factor’s ‘main effect’ (i.e. main effects are not aliased by other main effects or two-factor effects). OACDs can also be used to study interaction terms and quadratic effects, unlike CCDs and SCDs which provide no information on interaction because each axial point contains only one nonzero component. Additionally, the OACD allows for cross-validation of results as multiple analyses can be performed separately on the same data set. Separate analyses can be performed on the two-level fractional factorial design to estimate linear and bi-linear effects, on the three-level orthogonal design to estimate linear and quadratic effects, and finally on the entire data set to estimate all linear, bi-linear and quadratic effects. Cross-validation between these three models estimates can confirm the reliability of the data obtained and the appropriateness of the model selected.

*Drug dose selection*

The highest dose, referred to as Dose 2, corresponds to effective dose (ED) of approximately 25% inhibition versus control (ED_25_). This drug dose is limited to avoid having any drug with a dominating single drug effect that would mask the effects of drug interactions. The lower drug dose, Dose 1, is selected in such a manner to enable the identification of potential drug-drug interactions and was maintained at a constant ratio as compared to Dose 2 for all drugs. As we have included 10 drugs in our optimization, a linear addition of the efficacy of the ten drugs would result in approximately 100% cell death if each compound contributes a maximum of 10% inhibitory effect. At the same time, Dose 1 should still be in the effective dose range to allow its interplay with other drugs. The lower dose was therefore selected to have an efficacy between EC_5_ and EC_10_. Finally, Dose 0 signified that no drug was added.

**Supplementary Figures**

**Supplementary Figure 1.**


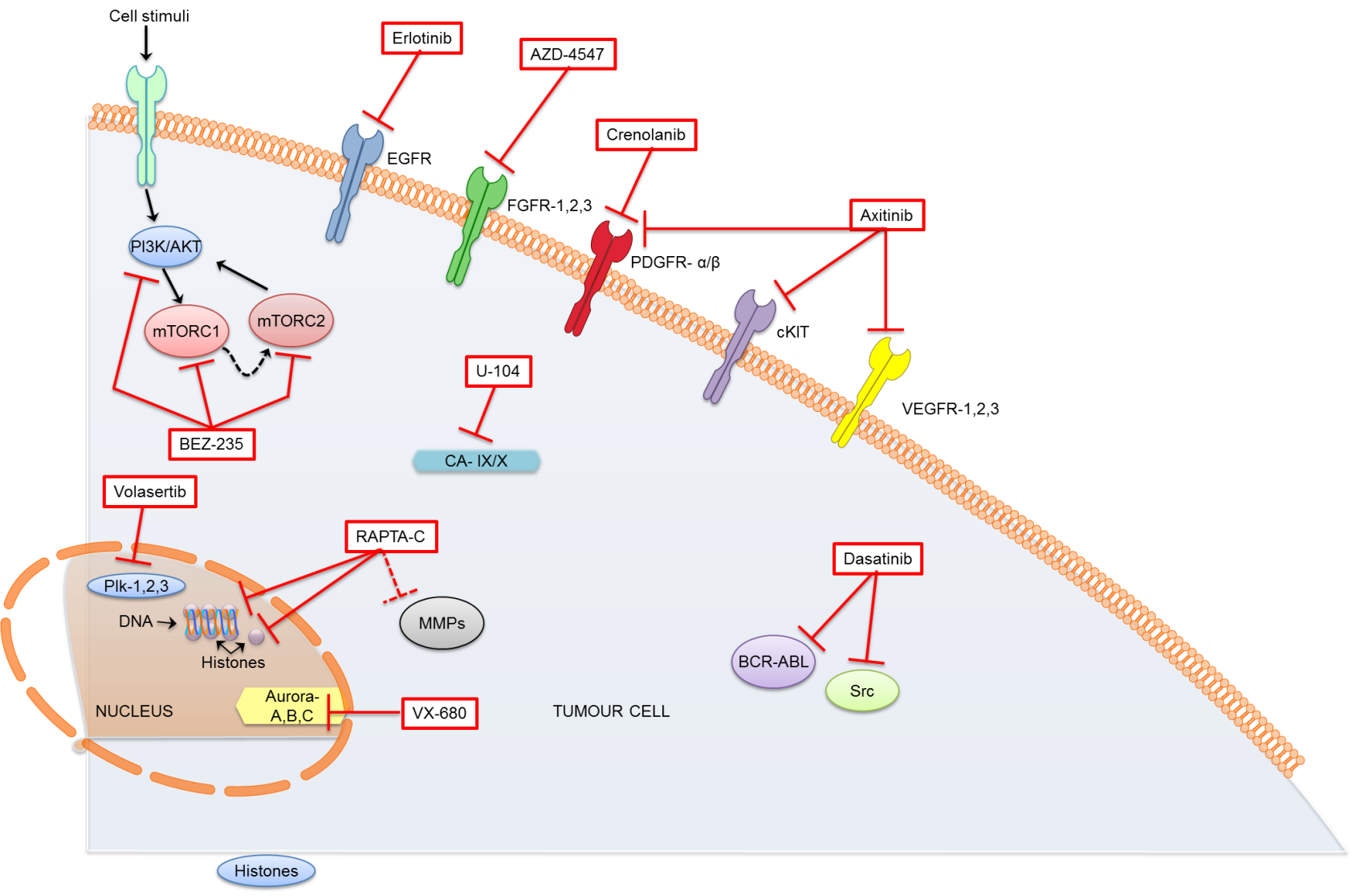


**Supplementary Figure 1**: Scheme of ten drugs covering a broad targeting spectrum in a cell: **axitinib** (VEGFR’s, PDGFR and c-KIT [^2^](#_ENREF_2)); **erlotinib** (EGFR inhibitor , that binds to transforming growth factor alpha (TGF-α)); **RAPTA-C** (chromatin inactivation [^5-7^](#_ENREF_5)); **BEZ-235** (inhibitor of both of the main protein complexes of mTOR (mechanistic target of rapamycin), mTORC1 and mTORC2 [^8^](#_ENREF_8)); **volasertib** (inhibitor of PLK- polo-like kinase, which is found in the nuclei of dividing cells, and controls multiple stages of cell cycle and division [^9^](#_ENREF_9)); **dasatinib** (inhibitor of BCR/ABL and Src, binding both active and inactive forms of ABL kinase[^10^](#_ENREF_10)); **VX-680** (inhibitor of Aurora A, B, C, which play a role in mitosis and meiosis during proliferation (max between G2 to M phase) [^11^](#_ENREF_11); **U-104** (inhibitor of carbonic anhydrase IX/X inhibitor II. CAIX expression is regulated by VHL protein, and VHL mutation loss is associated with clear cell RCC, so CAIX expression is related to clear cell RCC ); **AZD4547** (TKI targeting FGFR 1-3 and also showing a weak activity against FGFR4 [^14^](#_ENREF_14)); **crenolanib** (specific PDGFR inhibitor[^15^](#_ENREF_15)).

**Supplementary Figure 2.**


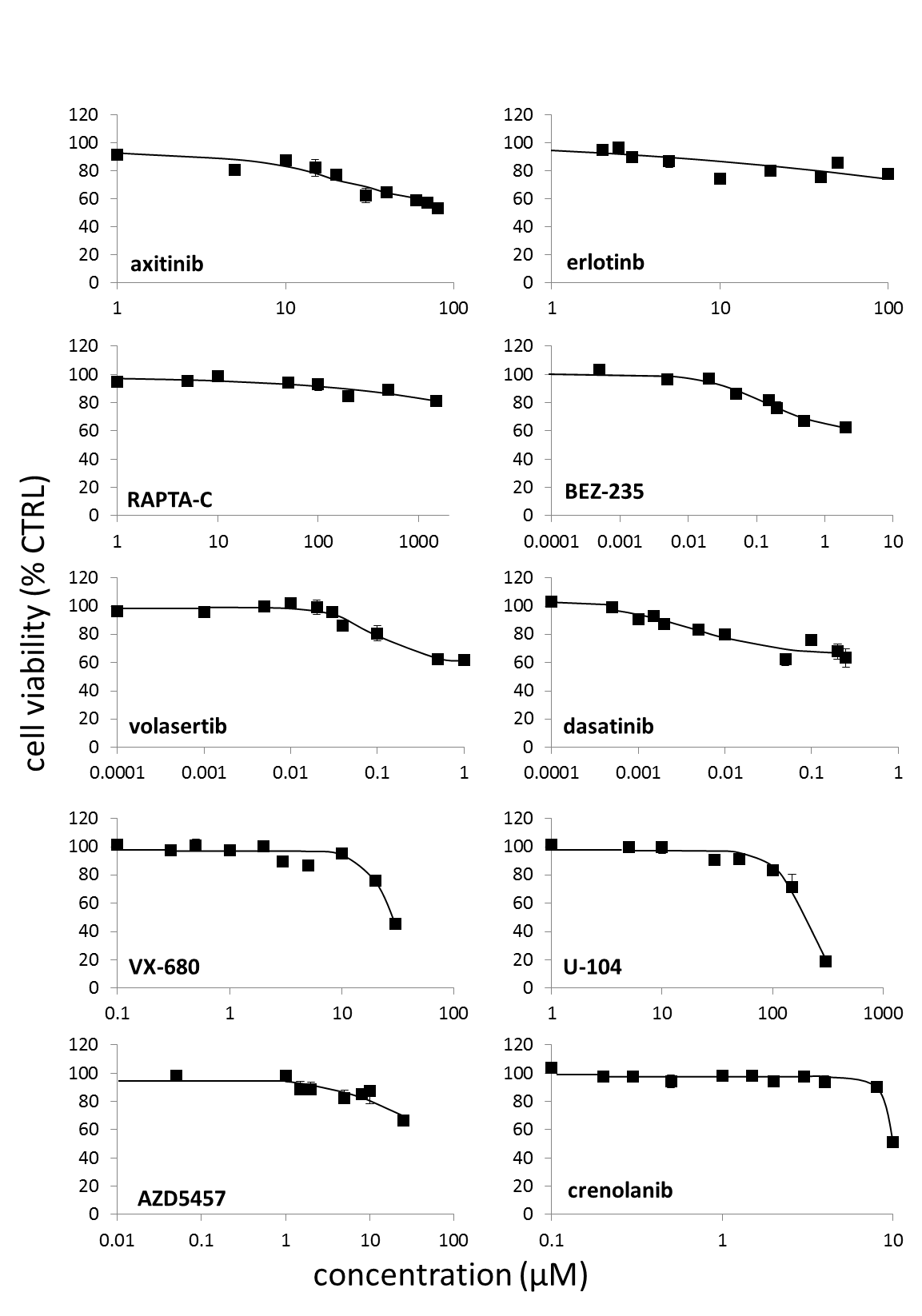


**Supplementary Figure 2**. Dose-response curves for each of the ten compounds on the inhibition of cell viability in the 768-O cell line. Points represent the average of at least two independent experiments, each performed in triplicate, error bars represent the standard error of mean (SEM).

**Supplementary Table 1**.

| **Combinations** | **Drug 1** | **Drug 2** | **Drug 3** | **Drug 4** | **Drug 5** | **Drug 6** | **Drug 7** | **Drug 8** | **Drug 9** | **Drug 10** |
| --- | --- | --- | --- | --- | --- | --- | --- | --- | --- | --- |
| **1** | 2 | 2 | 2 | 2 | 2 | 2 | 2 | 2 | 2 | 2 |
| **2** | 2 | 2 | 2 | 2 | 2 | 0 | 2 | 0 | 0 | 0 |
| **3** | 2 | 2 | 2 | 2 | 0 | 2 | 0 | 2 | 2 | 0 |
| **4** | 2 | 2 | 2 | 2 | 0 | 0 | 0 | 0 | 0 | 2 |
| **5** | 2 | 2 | 2 | 0 | 2 | 2 | 0 | 2 | 0 | 2 |
| **6** | 2 | 2 | 2 | 0 | 2 | 0 | 0 | 0 | 2 | 0 |
| **7** | 2 | 2 | 2 | 0 | 0 | 2 | 2 | 2 | 0 | 0 |
| **8** | 2 | 2 | 2 | 0 | 0 | 0 | 2 | 0 | 2 | 2 |
| **9** | 2 | 2 | 0 | 2 | 2 | 2 | 0 | 0 | 2 | 2 |
| **10** | 2 | 2 | 0 | 2 | 2 | 0 | 0 | 2 | 0 | 0 |
| **11** | 2 | 2 | 0 | 2 | 0 | 2 | 2 | 0 | 2 | 0 |
| **12** | 2 | 2 | 0 | 2 | 0 | 0 | 2 | 2 | 0 | 2 |
| **13** | 2 | 2 | 0 | 0 | 2 | 2 | 2 | 0 | 0 | 2 |
| **14** | 2 | 2 | 0 | 0 | 2 | 0 | 2 | 2 | 2 | 0 |
| **15** | 2 | 2 | 0 | 0 | 0 | 2 | 0 | 0 | 0 | 0 |
| **16** | 2 | 2 | 0 | 0 | 0 | 0 | 0 | 2 | 2 | 2 |
| **17** | 2 | 0 | 2 | 2 | 2 | 2 | 0 | 0 | 2 | 0 |
| **18** | 2 | 0 | 2 | 2 | 2 | 0 | 0 | 2 | 0 | 2 |
| **19** | 2 | 0 | 2 | 2 | 0 | 2 | 2 | 0 | 2 | 2 |
| **20** | 2 | 0 | 2 | 2 | 0 | 0 | 2 | 2 | 0 | 0 |
| **21** | 2 | 0 | 2 | 0 | 2 | 2 | 2 | 0 | 0 | 0 |
| **22** | 2 | 0 | 2 | 0 | 2 | 0 | 2 | 2 | 2 | 2 |
| **23** | 2 | 0 | 2 | 0 | 0 | 2 | 0 | 0 | 0 | 2 |
| **24** | 2 | 0 | 2 | 0 | 0 | 0 | 0 | 2 | 2 | 0 |
| **25** | 2 | 0 | 0 | 2 | 2 | 2 | 2 | 2 | 2 | 0 |
| **26** | 2 | 0 | 0 | 2 | 2 | 0 | 2 | 0 | 0 | 2 |
| **27** | 2 | 0 | 0 | 2 | 0 | 2 | 0 | 2 | 2 | 2 |
| **28** | 2 | 0 | 0 | 2 | 0 | 0 | 0 | 0 | 0 | 0 |
| **29** | 2 | 0 | 0 | 0 | 2 | 2 | 0 | 2 | 0 | 0 |
| **30** | 2 | 0 | 0 | 0 | 2 | 0 | 0 | 0 | 2 | 2 |
| **31** | 2 | 0 | 0 | 0 | 0 | 2 | 2 | 2 | 0 | 2 |
| **32** | 2 | 0 | 0 | 0 | 0 | 0 | 2 | 0 | 2 | 0 |
| **33** | 0 | 2 | 2 | 2 | 2 | 2 | 0 | 0 | 0 | 0 |
| **34** | 0 | 2 | 2 | 2 | 2 | 0 | 0 | 2 | 2 | 2 |
| **35** | 0 | 2 | 2 | 2 | 0 | 2 | 2 | 0 | 0 | 2 |
| **36** | 0 | 2 | 2 | 2 | 0 | 0 | 2 | 2 | 2 | 0 |
| **37** | 0 | 2 | 2 | 0 | 2 | 2 | 2 | 0 | 2 | 0 |
| **38** | 0 | 2 | 2 | 0 | 2 | 0 | 2 | 2 | 0 | 2 |
| **39** | 0 | 2 | 2 | 0 | 0 | 2 | 0 | 0 | 2 | 2 |
| **40** | 0 | 2 | 2 | 0 | 0 | 0 | 0 | 2 | 0 | 0 |
| **41** | 0 | 2 | 0 | 2 | 2 | 2 | 2 | 2 | 0 | 0 |
| **42** | 0 | 2 | 0 | 2 | 2 | 0 | 2 | 0 | 2 | 2 |
| **43** | 0 | 2 | 0 | 2 | 0 | 2 | 0 | 2 | 0 | 2 |
| **44** | 0 | 2 | 0 | 2 | 0 | 0 | 0 | 0 | 2 | 0 |
| **45** | 0 | 2 | 0 | 0 | 2 | 2 | 0 | 2 | 2 | 0 |
| **46** | 0 | 2 | 0 | 0 | 2 | 0 | 0 | 0 | 0 | 2 |
| **47** | 0 | 2 | 0 | 0 | 0 | 2 | 2 | 2 | 2 | 2 |
| **48** | 0 | 2 | 0 | 0 | 0 | 0 | 2 | 0 | 0 | 0 |
| **49** | 0 | 0 | 2 | 2 | 2 | 2 | 2 | 2 | 0 | 2 |
| **50** | 0 | 0 | 2 | 2 | 2 | 0 | 2 | 0 | 2 | 0 |
| **51** | 0 | 0 | 2 | 2 | 0 | 2 | 0 | 2 | 0 | 0 |
| **52** | 0 | 0 | 2 | 2 | 0 | 0 | 0 | 0 | 2 | 2 |
| **53** | 0 | 0 | 2 | 0 | 2 | 2 | 0 | 2 | 2 | 2 |
| **54** | 0 | 0 | 2 | 0 | 2 | 0 | 0 | 0 | 0 | 0 |
| **55** | 0 | 0 | 2 | 0 | 0 | 2 | 2 | 2 | 2 | 0 |
| **56** | 0 | 0 | 2 | 0 | 0 | 0 | 2 | 0 | 0 | 2 |
| **57** | 0 | 0 | 0 | 2 | 2 | 2 | 0 | 0 | 0 | 2 |
| **58** | 0 | 0 | 0 | 2 | 2 | 0 | 0 | 2 | 2 | 0 |
| **59** | 0 | 0 | 0 | 2 | 0 | 2 | 2 | 0 | 0 | 0 |
| **60** | 0 | 0 | 0 | 2 | 0 | 0 | 2 | 2 | 2 | 2 |
| **61** | 0 | 0 | 0 | 0 | 2 | 2 | 2 | 0 | 2 | 2 |
| **62** | 0 | 0 | 0 | 0 | 2 | 0 | 2 | 2 | 0 | 0 |
| **63** | 0 | 0 | 0 | 0 | 0 | 2 | 0 | 0 | 2 | 0 |
| **64** | 0 | 0 | 0 | 0 | 0 | 0 | 0 | 2 | 0 | 0 |
| **65** | 0 | 0 | 0 | 0 | 0 | 0 | 0 | 0 | 0 | 0 |
| **66** | 0 | 2 | 2 | 0 | 1 | 1 | 0 | 1 | 2 | 0 |
| **67** | 0 | 1 | 1 | 0 | 2 | 2 | 0 | 2 | 1 | 0 |
| **68** | 2 | 1 | 1 | 1 | 0 | 1 | 1 | 0 | 2 | 0 |
| **69** | 2 | 0 | 0 | 1 | 1 | 2 | 1 | 1 | 1 | 0 |
| **70** | 2 | 2 | 2 | 1 | 2 | 0 | 1 | 2 | 0 | 0 |
| **71** | 1 | 2 | 2 | 2 | 0 | 2 | 2 | 0 | 1 | 0 |
| **72** | 1 | 1 | 1 | 2 | 1 | 0 | 2 | 1 | 0 | 0 |
| **73** | 1 | 0 | 0 | 2 | 2 | 1 | 2 | 2 | 2 | 0 |
| **74** | 1 | 1 | 0 | 0 | 0 | 1 | 1 | 1 | 1 | 1 |
| **75** | 1 | 0 | 2 | 0 | 1 | 2 | 1 | 2 | 0 | 1 |
| **76** | 1 | 2 | 1 | 0 | 2 | 0 | 1 | 0 | 2 | 1 |
| **77** | 0 | 2 | 1 | 1 | 0 | 2 | 2 | 1 | 0 | 1 |
| **78** | 0 | 1 | 0 | 1 | 1 | 0 | 2 | 2 | 2 | 1 |
| **79** | 0 | 0 | 2 | 1 | 2 | 1 | 2 | 0 | 1 | 1 |
| **80** | 2 | 0 | 2 | 2 | 0 | 0 | 0 | 1 | 2 | 1 |
| **81** | 2 | 2 | 1 | 2 | 1 | 1 | 0 | 2 | 1 | 1 |
| **82** | 2 | 1 | 0 | 2 | 2 | 2 | 0 | 0 | 0 | 1 |
| **83** | 2 | 2 | 0 | 0 | 0 | 2 | 2 | 2 | 2 | 2 |
| **84** | 2 | 1 | 2 | 0 | 1 | 0 | 2 | 0 | 1 | 2 |
| **85** | 2 | 0 | 1 | 0 | 2 | 1 | 2 | 1 | 0 | 2 |
| **86** | 1 | 0 | 1 | 1 | 0 | 0 | 0 | 2 | 1 | 2 |
| **87** | 1 | 2 | 0 | 1 | 1 | 1 | 0 | 0 | 0 | 2 |
| **88** | 1 | 1 | 2 | 1 | 2 | 2 | 0 | 1 | 2 | 2 |
| **89** | 0 | 1 | 2 | 2 | 0 | 1 | 1 | 2 | 0 | 2 |
| **90** | 0 | 0 | 1 | 2 | 1 | 2 | 1 | 0 | 2 | 2 |
| **91** | 0 | 2 | 0 | 2 | 2 | 0 | 1 | 1 | 1 | 2 |

**Supplementary Table 1:** Ninety-one drug combinations based on a 3-level OACD matrix used in search 1. The 10 most (green) and least (orange) effective drug combinations are indicated.

**Supplementary Table 2**.

| **Combination** | **Drug 1** | **Drug 2** | **Drug 3** | **Drug 4** | **Drug 5** | **Drug 6** | **Drug 7** |
| --- | --- | --- | --- | --- | --- | --- | --- |
| **1** | 2 | 2 | 2 | 2 | 2 | 2 | 2 |
| **2** | 2 | 2 | 2 | 2 | 0 | 2 | 0 |
| **3** | 2 | 2 | 2 | 0 | 2 | 0 | 2 |
| **4** | 2 | 2 | 2 | 0 | 0 | 0 | 0 |
| **5** | 2 | 2 | 0 | 2 | 2 | 0 | 2 |
| **6** | 2 | 2 | 0 | 2 | 0 | 0 | 0 |
| **7** | 2 | 2 | 0 | 0 | 2 | 2 | 2 |
| **8** | 2 | 2 | 0 | 0 | 0 | 2 | 0 |
| **9** | 2 | 0 | 2 | 2 | 2 | 0 | 0 |
| **10** | 2 | 0 | 2 | 2 | 0 | 0 | 2 |
| **11** | 2 | 0 | 2 | 0 | 2 | 2 | 0 |
| **12** | 2 | 0 | 2 | 0 | 0 | 2 | 2 |
| **13** | 2 | 0 | 0 | 2 | 2 | 2 | 0 |
| **14** | 2 | 0 | 0 | 2 | 0 | 2 | 2 |
| **15** | 2 | 0 | 0 | 0 | 2 | 0 | 0 |
| **16** | 2 | 0 | 0 | 0 | 0 | 0 | 2 |
| **17** | 0 | 2 | 2 | 2 | 2 | 0 | 0 |
| **18** | 0 | 2 | 2 | 2 | 0 | 0 | 2 |
| **19** | 0 | 2 | 2 | 0 | 2 | 2 | 0 |
| **20** | 0 | 2 | 2 | 0 | 0 | 2 | 2 |
| **21** | 0 | 2 | 0 | 2 | 2 | 2 | 0 |
| **22** | 0 | 2 | 0 | 2 | 0 | 2 | 2 |
| **23** | 0 | 2 | 0 | 0 | 2 | 0 | 0 |
| **24** | 0 | 2 | 0 | 0 | 0 | 0 | 2 |
| **25** | 0 | 0 | 2 | 2 | 2 | 2 | 2 |
| **26** | 0 | 0 | 2 | 2 | 0 | 2 | 0 |
| **27** | 0 | 0 | 2 | 0 | 2 | 0 | 2 |
| **28** | 0 | 0 | 2 | 0 | 0 | 0 | 0 |
| **29** | 0 | 0 | 0 | 2 | 2 | 0 | 2 |
| **30** | 0 | 0 | 0 | 2 | 0 | 0 | 0 |
| **31** | 0 | 0 | 0 | 0 | 2 | 2 | 2 |
| **32** | 0 | 0 | 0 | 0 | 0 | 2 | 0 |
| **33** | 0 | 0 | 0 | 0 | 0 | 0 | 0 |
| **34** | 0 | 1 | 1 | 1 | 1 | 1 | 1 |
| **35** | 0 | 2 | 2 | 2 | 2 | 2 | 2 |
| **36** | 1 | 0 | 1 | 0 | 1 | 2 | 2 |
| **37** | 1 | 1 | 2 | 1 | 2 | 0 | 0 |
| **38** | 1 | 2 | 0 | 2 | 0 | 1 | 1 |
| **39** | 2 | 0 | 2 | 1 | 0 | 2 | 1 |
| **40** | 2 | 1 | 0 | 2 | 1 | 0 | 2 |
| **41** | 2 | 2 | 1 | 0 | 2 | 1 | 0 |
| **42** | 0 | 0 | 1 | 2 | 2 | 0 | 1 |
| **43** | 0 | 1 | 2 | 0 | 0 | 1 | 2 |
| **44** | 0 | 2 | 0 | 1 | 1 | 2 | 0 |
| **45** | 1 | 0 | 0 | 1 | 2 | 1 | 2 |
| **46** | 1 | 1 | 1 | 2 | 0 | 2 | 0 |
| **47** | 1 | 2 | 2 | 0 | 1 | 0 | 1 |
| **48** | 2 | 0 | 2 | 2 | 1 | 1 | 0 |
| **49** | 2 | 1 | 0 | 0 | 2 | 2 | 1 |
| **50** | 2 | 2 | 1 | 1 | 0 | 0 | 2 |

**Supplementary Table 2.** Design matrices contained 50 (Search 2.1) and 25 (Search 2.2) drug combinations. The 10 most and least effective combinations are indicated in green and orange, respectively, based on the results of Search 2.1. The 10 most (green) and least (orange) effective drug combinations are indicated.

**Supplementary Table 3.**

| **Combinations** | **Drug 1** | **Drug 2** | **Drug 3** | **Drug 4** |
| --- | --- | --- | --- | --- |
| **1** | 2 | 2 | 2 | 2 |
| **2** | 2 | 2 | 2 | 0 |
| **3** | 2 | 2 | 0 | 2 |
| **4** | 2 | 2 | 0 | 0 |
| **5** | 2 | 0 | 2 | 2 |
| **6** | 2 | 0 | 2 | 0 |
| **7** | 2 | 0 | 0 | 2 |
| **8** | 2 | 0 | 0 | 0 |
| **9** | 0 | 2 | 2 | 2 |
| **10** | 0 | 2 | 2 | 0 |
| **11** | 0 | 2 | 0 | 2 |
| **12** | 0 | 2 | 0 | 0 |
| **13** | 0 | 0 | 2 | 2 |
| **14** | 0 | 0 | 2 | 0 |
| **15** | 0 | 0 | 0 | 2 |
| **16** | 0 | 0 | 0 | 0 |
| **17** | 0 | 0 | 0 | 0 |
| **18** | 0 | 1 | 1 | 2 |
| **19** | 0 | 2 | 2 | 1 |
| **20** | 1 | 0 | 1 | 1 |
| **21** | 1 | 1 | 2 | 0 |
| **22** | 1 | 2 | 0 | 2 |
| **23** | 2 | 0 | 2 | 2 |
| **24** | 2 | 1 | 0 | 1 |
| **25** | 2 | 2 | 1 | 0 |
|  |  |  |  |  |

**Supplementary Table 3:** Search 3 was performed by testing 25 drug combination design matrix**.** The 10 most (green) and least (orange) effective drug combinations are indicated in, based on the results of Search 2.2.

**Supplementary References**

1 Xu, H., Jaynes, Jessica, Ding, Xianting. Combinig two-level and three-level orthogonal arrays for factor screening and response surface exploration. *Statistica Sinica* **24**, 269-289 (2014).

2 Gross-Goupil, M., Francois, L., Quivy, A. & Ravaud, A. Axitinib: A Review of its Safety and Efficacy in the Treatment of Adults with Advanced Renal Cell Carcinoma. *Clinical Medicine Insights. Oncology* **7**, 269-277, (2013).

3 Ling, J. *et al.* Metabolism and excretion of erlotinib, a small molecule inhibitor of epidermal growth factor receptor tyrosine kinase, in healthy male volunteers. *Drug Metab Dispos* **34**, 420-426, (2006).

4 Bukowski, R. M. *et al.* Randomized phase II study of erlotinib combined with bevacizumab compared with bevacizumab alone in metastatic renal cell cancer. *J Clin Oncol* **25**, 4536-4541, doi:10.1200/JCO.2007.11.5154 (2007).

5 Nowak-Sliwinska, P. *et al.* Organometallic ruthenium(II) arene compounds with antiangiogenic activity. *J Med Chem* **54**, 3895-3902, (2011).

6 Rugo, H. S., Stopeck, A. & Joy, A. A. A randomized, double-blind phase II study of the oral tyrosine kinase inhibitor (TKI) axitinib (AG-013736) in combination with docetaxel (DOC) compared to DOC plus placebo (PL) in metastatic breast cancer (MBC). *J Clin Oncol* **25(18 supl)**, 32 (2007).

7 Sumi, T. *et al.* Expression of matrix metalloproteinases 7 and 2 in human renal cell carcinoma. *Oncol Rep* **10**, 567-570 (2003).

8 Cho, D. C. *et al.* The efficacy of the novel dual PI3-kinase/mTOR inhibitor NVP-BEZ235 compared with rapamycin in renal cell carcinoma. *Clin Cancer Res* **16**, 3628-3638, (2010).

9 Schoffski, P. *et al.* A phase I, dose-escalation study of the novel Polo-like kinase inhibitor volasertib (BI 6727) in patients with advanced solid tumours. *Eur J Cancer* **48**, 179-186, (2012).

10 Lombardo, L. J. *et al.* Discovery of N-(2-chloro-6-methyl- phenyl)-2-(6-(4-(2-hydroxyethyl)- piperazin-1-yl)-2-methylpyrimidin-4- ylamino)thiazole-5-carboxamide (BMS-354825), a dual Src/Abl kinase inhibitor with potent antitumor activity in preclinical assays. *J Med Chem* **47**, 6658-6661, (2004).

11 Li, Y. *et al.* VX680/MK-0457, a potent and selective Aurora kinase inhibitor, targets both tumor and endothelial cells in clear cell renal cell carcinoma. *Am J Transl Res* **2**, 296-308 (2010).

12 Stillebroer, A., Mulders, P., Boerman, O., Oyen, W. & Oosterwijk, E. Carbonic anhydrase IX in renal cell carcinoma: implications for prognosis, diagnosis, and therapy. *Eur Urol* **58**, 75-83, (2010).

13 Al-Ahmadie, H. A. *et al.* Carbonic anhydrase IX expression in clear cell renal cell carcinoma: an immunohistochemical study comparing 2 antibodies. *Am J Surg Pathol* **32**, 377-382, (2008).

14 Zhang, J. *et al.* Translating the therapeutic potential of AZD4547 in FGFR1-amplified non-small cell lung cancer through the use of patient-derived tumor xenograft models. *Clin Cancer Res* **18**, 6658-6667, (2012).

15 Heinrich, M. C. *et al.* Crenolanib inhibits the drug-resistant PDGFRA D842V mutation associated with imatinib-resistant gastrointestinal stromal tumors. *Clin Cancer Res* **18**, 4375-4384, (2012).
